# Supplementary figures and images for: Moderation analysis of subjective well-being, self-efficacy, and academic performance of 4th grade children in Russia
Source: PLoS One. 2026 Feb 2;21(2):e0341318. doi: 10.1371/journal.pone.0341318 (PMC12863477; doi:10.1371/journal.pone.0341318)

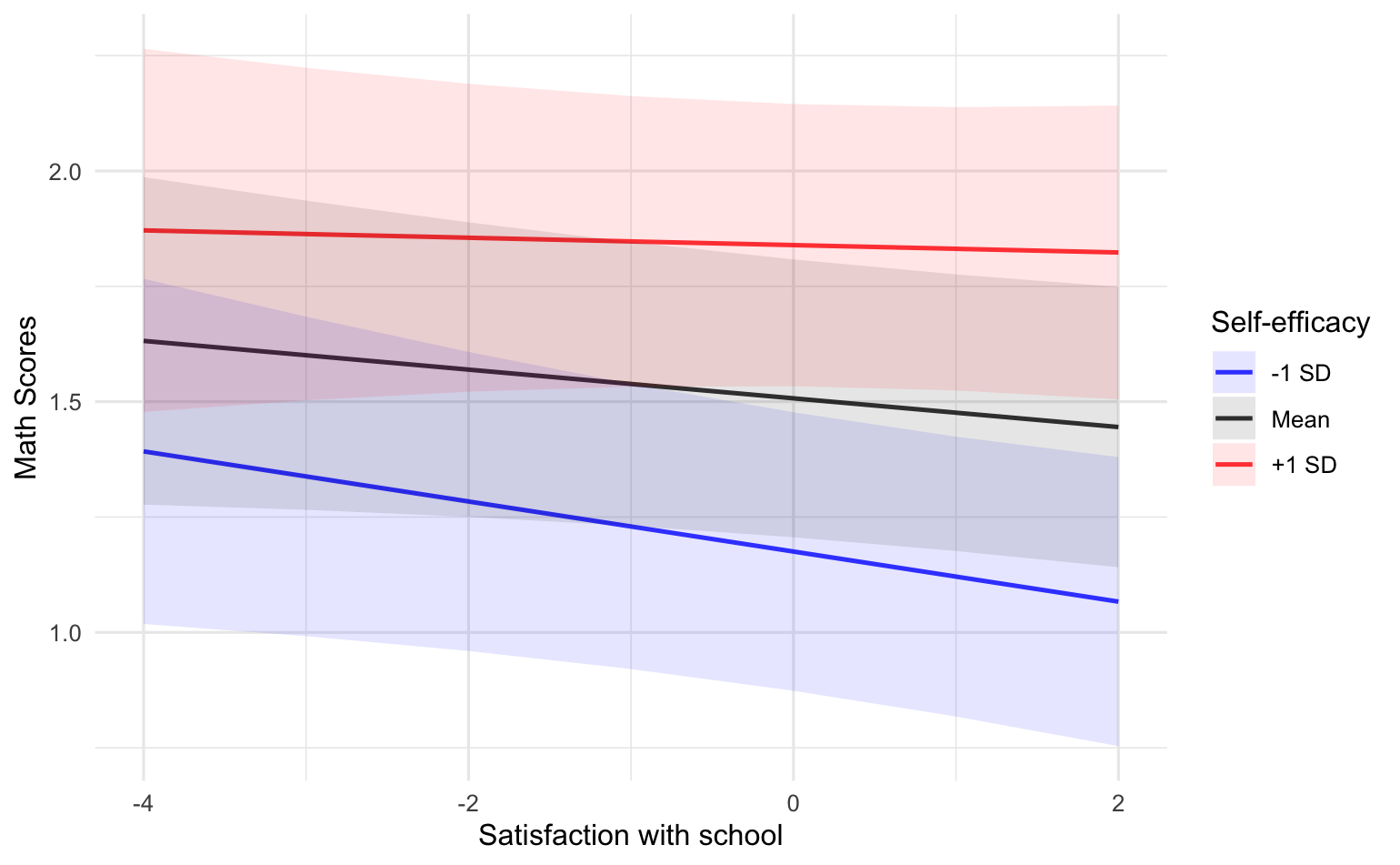

Supplement: S1 Fig — (TIFF) [file pone.0341318.s001.tiff]

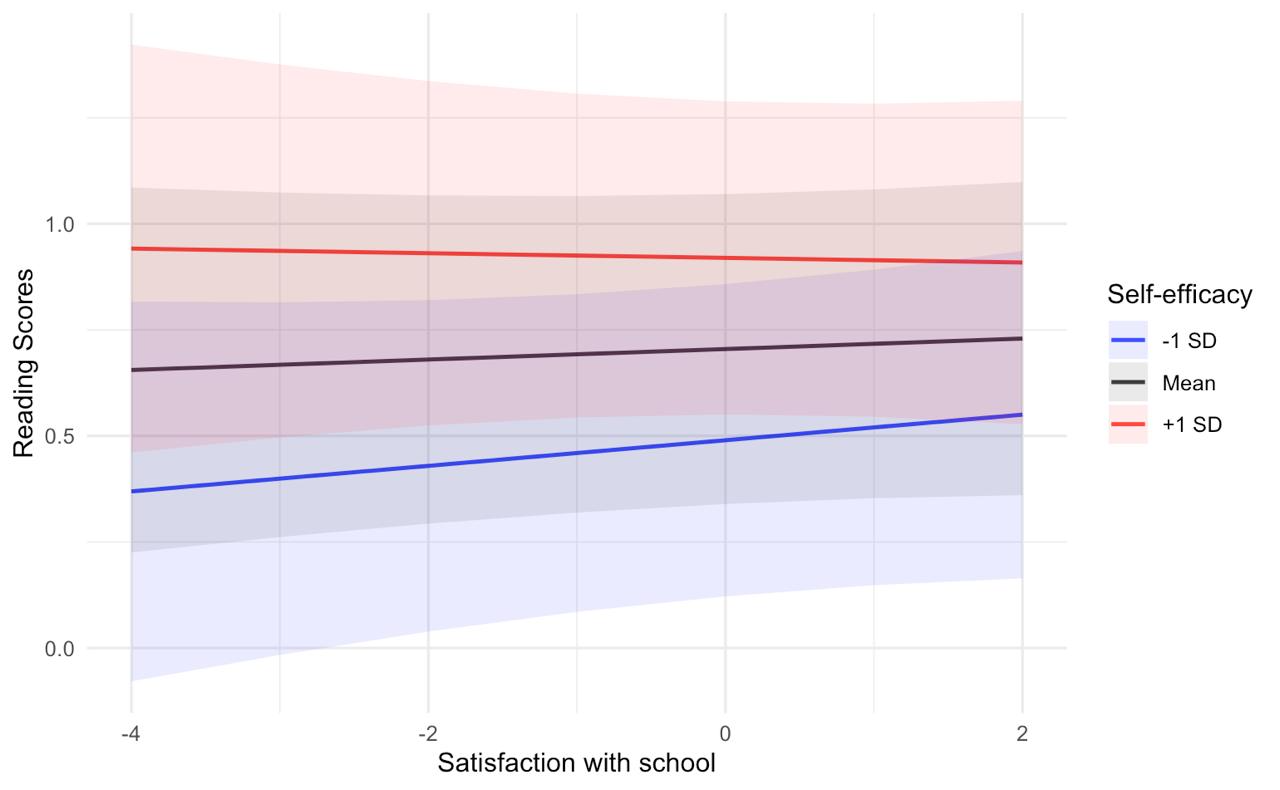

Supplement: S2 Fig — (TIFF) [file pone.0341318.s002.tiff]

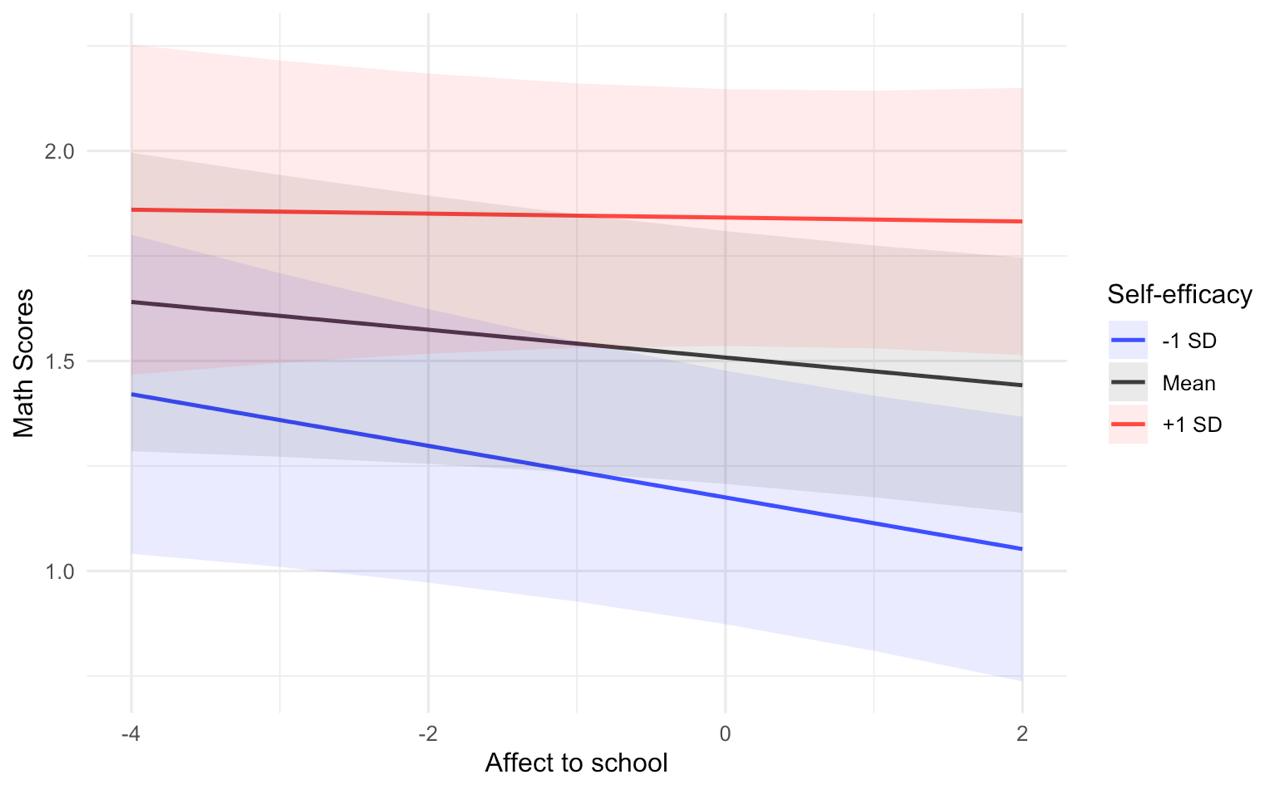

Supplement: S3 Fig — (TIFF) [file pone.0341318.s003.tiff]

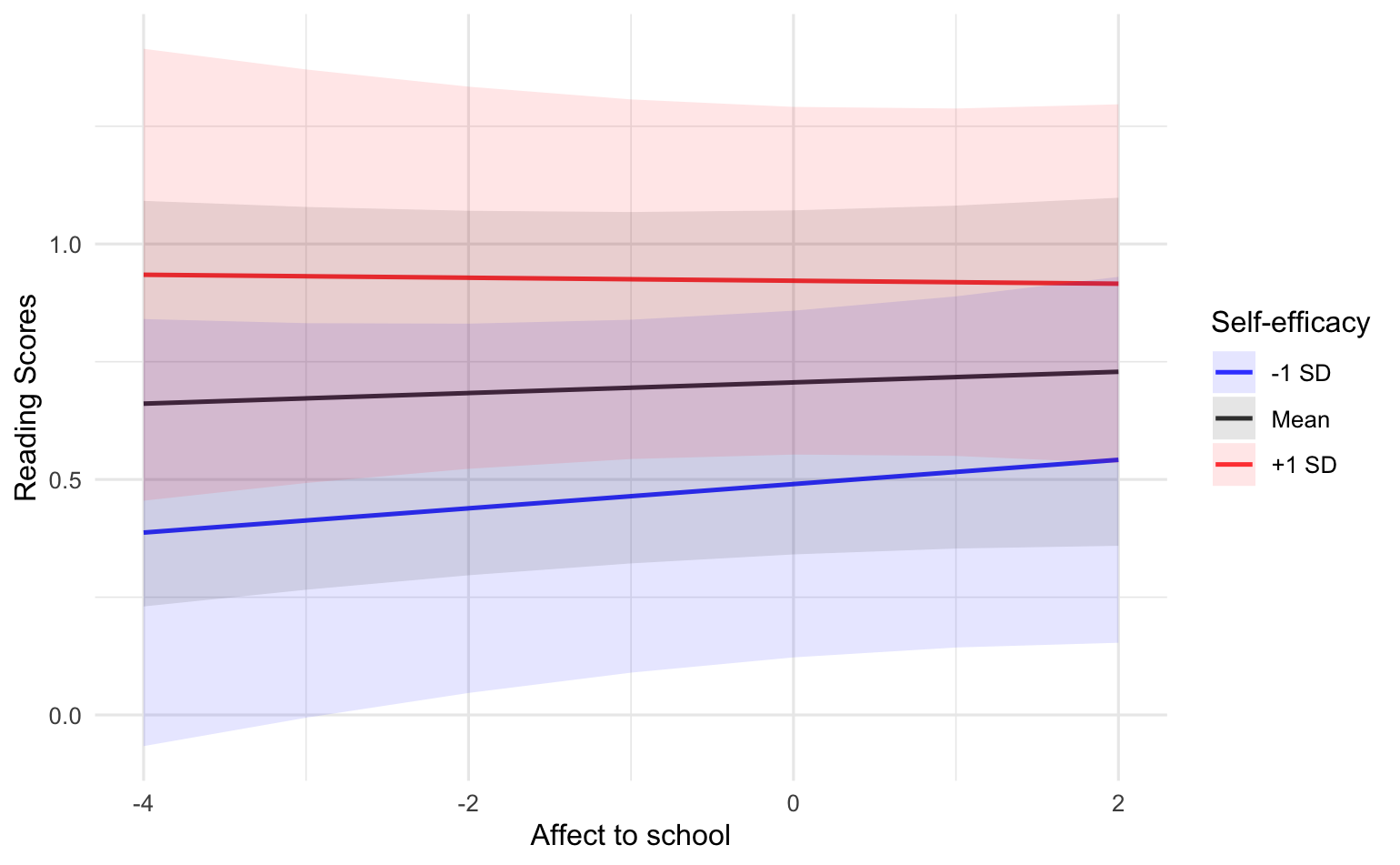

Supplement: S4 Fig — (TIFF) [file pone.0341318.s004.tiff]
