# Supplementary material for: Moderation analysis of subjective well-being, self-efficacy, and academic performance of 4th grade children in Russia
Source: PLoS One. 2026 Feb 2;21(2):e0341318. doi: 10.1371/journal.pone.0341318 (PMC12863477; doi:10.1371/journal.pone.0341318)
Supplement: Attachment — Submitted filename: Response to Reviewers.docx [file pone.0341318.s007.docx]

Letter of Revisions

This letter of revisions accompanies the revised manuscript *Moderation analysis of subjective well-being, self-efficacy, and academic performance of 4^th^ grade children in Russia* in response to the Reviewer’s 1 and 3 feedback as well as to editorial comments. We would like to express our deepest gratitude to anonymous reviewers who provided very detailed and constructive feedback on this paper. This letter of revisions provides the detailed response to each comment.

Based on the feedback, we revised the abstract, created separate introduction and literature, and highlighted the research gaps, novelty, and scientific significance of this study in the introduction and discussion sections. All the revisions to the text are represented in the blue font.

In this section, we are responding to each of the comments provided by Reviewer 1:

1. **Abstract:** The abstract effectively introduces the aim of the study, which is to explore how subjective well-being and domain-specific self-efficacy predict academic achievement in mathematics and reading among elementary school students in Russia. However, the abstract would benefit from being more concise. Specific numerical results (e.g., strength of relationships, effect sizes) are missing, and the conclusion is vague. Including a sentence on practical implications could enhance its scholarly value and clarity. – Thank you for your feedback. We tried to keep the abstract concise, yet added the numerical results and an example of a specific practical implication (p. 2).
2. **The keywords:** The keywords selected, “subjective well-being,” “self-efficacy,” “mathematics,” “reading,” “elementary school,” and “moderation,” are broadly relevant to the study’s scope. However, the inclusion of terms like “gender differences” and “academic achievement” would strengthen discoverability in academic databases. – Thank you for the suggestion. We added the additional keywords (p. 2).
3. **Introduction:** The introduction presents a compelling rationale for the research by highlighting the growing emphasis on students’ well-being alongside academic achievement. It provides a well-synthesized background on subjective well-being and self-efficacy, referring to both international and Russian educational contexts. The discussion of prior inconsistent findings helps justify the current research questions. Nevertheless, the introduction would benefit from improved logical flow and more emphasis on the novelty of this study, namely, the focus on elementary students in Russia using moderation analysis. Additionally, some opening paragraphs are slightly philosophical and could be tightened to maintain academic focus. – Thank you for your suggestion. We decided to create introduction as a separate section, where we describe the research gap, novelty, and contributions of this study (p. 3).
4. **Literature Review:** Although not presented as a distinct section, the literature review is embedded throughout the introduction and early pages. It provides a good overview of key concepts, such as subjective well-being and domain-specific self-efficacy, and discusses findings from major meta-analyses. The review includes important international sources and situates the study within the OECD’s broader educational goals. However, there is a limited critical analysis of Russian-specific literature, and some referenced studies are outdated or not fully detailed (e.g., missing author names). Structuring this into a standalone literature review section with clearer subsections and more recent sources would improve the depth and clarity of the theoretical foundation. - Thank you for your suggestion! We separated the introduction from the literature review and added relevant studies on subjective well-being (p. 5) and self-efficacy (p.8) conducted in Russia. These were the only studies which were conducted in Russia and were done rigorously enough to include them as part of the literature review section. As can be seen, there are a handful studies in general, and only a couple of studies conducted in upper elementary schools. The missing authors’ names in the references was the attempt to blind the manuscript for the review process, since most of them are conducted by the authors of this paper within the last five years. We added all authors’ names at this point (pp. 25 – 27).
5. **Methodology:** The methodology section is detailed and logically structured. The study design is part of a longitudinal project, although the current data is cross-sectional. Participants were drawn from 40 schools in central Russia, with a sample size of 1,962 fourth-grade students. The use of well-validated instruments to assess well-being and self-efficacy is commendable, and psychometric properties are reported. The statistical approach, including OLS regression and moderation analysis with interaction terms, is appropriate and carefully explained. However, **justification for using listwise deletion for missing data instead of more robust methods (like multiple imputation) is lacking**. Also, the rationale for not testing both types of self-efficacy in the same model due to high correlation could be better supported with variance inflation factor (VIF) data. Additional information about the sampling process and school selection would strengthen transparency. – Thank you for your suggestion. Information on the sampling procedures and criteria are added on p.9.

We acknowledge the reviewer's concern regarding the use of listwise deletion for handling missing data. Indeed, multiple imputation is considered a robust and preferred method in many contexts. However, our decision to apply listwise deletion was carefully considered based on the characteristics of our data and practical limitations. In our dataset, up to 13% of the values were missing in variables that underpin key constructs such as self-efficacy in math and reading, affect towards school, and satisfaction with school. Importantly, these missing values were due to students skipping specific questions based on which these constructs generated. Unfortunately, the dataset provides only limited auxiliary information beyond these key variables—namely students’ gender, outcome variables (test scores), and location. Multiple imputation requires appropriate auxiliary data that are predictive of both the missingness and the missing values themselves to produce reliable imputations (Graham, 2009). Given the limited auxiliary variables available, we opted for listwise deletion to preserve the validity and interpretability of our findings while acknowledging its limitations. We believe that this approach provides a cautious and justifiable handling of missing data in our study context. We added this information in footnotes on pp. 9-10 to make our decision transparent.

John W. Graham (2009). "Missing Data Analysis: Making It Work in the Real World"._Annual Review of Psychology, 60:549 576._<https://www.annualreviews.org/doi/10.1146/annurev.psych.58.110405.085530>

In terms of VIF, we included self-efficacy in math as a predictor only in models where math score was the outcome, and self-efficacy in reading only where reading score was the outcome, following the rationale that domain-specific self-efficacy most directly influences outcomes in the corresponding subject. We also chose not to include both satisfaction with school and affect to school in the same model due to their extremely high correlation (r=0,97). When included together, these variables yield a variance inflation factor (VIF) of 19, indicating severe multicollinearity, which compromises the stability and interpretability of regression coefficients.

6. **Discussion:** The discussion section appropriately interprets the study’s findings and connects them to prior research. It confirms that while subjective well-being is weakly associated with academic outcomes, self-efficacy plays a stronger and more consistent role. The authors provide insightful commentary on gender differences, noting that the moderation effect of self-efficacy is only significant for girls in math. They also suggest that fourth-grade children may not yet have fully developed cognitive abilities to assess their self-efficacy accurately. However, the discussion could be improved by integrating more socio-cultural context into gendered perceptions of math and reading. Additionally, the potential influence of classroom practices and teacher feedback on self-efficacy is mentioned in limitations but not explored deeply in the interpretation of findings. – Thank you for your comment. We added this information in the discussion section on p. 22 and in future research on p. 23.
7. **References:** The references cited are mostly recent and relevant, including key meta-analyses, foundational theories, and empirical studies on self-efficacy and well-being. However, there are inconsistencies in citation formatting, and some references are incomplete (e.g., anonymous or missing authors). The inclusion of more Russian-language or context-specific studies would enrich the regional relevance of the paper. – Thank you for your observation. We revised and edited the reference list in accordance with the Vancouver citation style.

In this section, we are responding to each of the comments provided by Reviewer #1:

1. General comment: It might be helpful if a direct link to the data is provided. The website has so much information and might not be the best for anyone interested to be looking for a pin in haystack. - Thank you for your observation. Unfortunately, there is no any direct link to the database to provide. Any interested person can find the dataset by entering the following number: 6.0036-2022 or by contacting the first author of this manuscript.
2. I believe the journal follows APA format and the citation and reference list needs to be in the format as well. - Thank you for your observation. However, the journal follows the Vancouver style. Therefore, we could not fix the formatting of citations and references. Please see more information by following this link: <https://journals.plos.org/plosone/s/submission-guidelines#loc-reference-style>
3. If OECD identified five domains, why is the study only using three domains? I wonder if the other two domains are not hedonistic as defined in the paper. Then it was scaled down to two school aspects and affects towards school. The reason for doing this is not very clear. – Thank you for your comment. We had to select the scales very carefully since this study is part of the longitudinal study and included a huge battery of tests and surveys. As a result, we aimed at keeping the surveys as short as possible but maintaining the rigor. Since these two scales showed high estimates in previous studies and we were interested in well-being in school, we decided to include just satisfaction with school and affect toward school scales.
4. At the last sentence before self-efficacy title, there is a repetition of study purpose. Thank you for your observation. We fixed it.
5. This statement is confusing, “the relationship between satisfaction with school and reading (Table 5, Column 2) becomes statistically not different from zero (p<0.05)” and “Finally, in all the models with school fixed effect (Tables 2–5, Column 4), the relationship between both types of wellbeing and both types of students’ outcomes is not statistically significant (p<0.05)”. I believe p < 0.05 means significant. The authors might want to look at the table again. – Thank you for your comment. We’ve deleted confusing “(p<0.05)” from the text.
6. Major Comment
   Did the authors use the validity and reliability in previous studies or the samples in this study? - Thank you for your question. Yes, the validity and reliability studies were conducted previously with all the instruments.

**Kanonire T, Federiakin DA, Uglanova IL. Multicomponent framework for students’ subjective well-being in elementary school. Sch Psychol. 2020 Sep;35(5):321-30. doi: 10.1037/spq0000397.**

**Akhmedjanova D. Domain-specific self-efficacy scales for elementary and middle school students. Psychol Russ-State Art. 2024;17(1):45 66. doi: 10.11621/pir.2024.0103.**

7. If the sub-domains of subjective wellbeing and self-efficacy have high correlation like that I wonder if they are truly two factors in the first place. Maybe the authors could provide some factor analysis results to substantiate these two factors. Stemming from the last comment, running those different analysis could negatively implicate the findings from the study and introduce error. I wonder if the authors will consider other analytical methods for this study. Latent based methods such as path analysis or others could be useful in this instance.– Thank you for your comment. Please see the papers cited above for the validity and reliability studies. Also, given the very high correlations between well-being subscales (r = 0.97) and between self-efficacy domains (r = 0.82), we opted not to include both subscales or both domains simultaneously in the same models to avoid issues of multicollinearity. By modeling these constructs separately, we aimed to provide clearer, more reliable estimates of their distinct associations and potential moderation effects. We added this text on p. 12 of the manuscript.
8. I wonder how the achievement tests are scored. If it is correct or wrong format, then kr-20 approach should be used instead of alpha and omega methods. I doubt how acceptable the internal consistency of reading test below 0.7 can be said to be acceptable. – Thank you for your comments, The achievement tests, both for math and reading were presented in the multiple-choice format. We provided some evidence of why the estimates were appropriate for further analyses. Specifically, we added the following text in the methods section (p.10). “Generally, the reliability estimates of > 0.70 are acceptable for conducting further analyses. However, some scholars establish acceptable reliability estimates at 0.65 [35] or at 0.6 [36]. Due to the reading test reliability estimates falling between 0.63 and 0.67, we concluded that the estimates were acceptable to conduct further analyses.”

**35. Taber KS. The use of Cronbach’s alpha when developing and reporting research instruments in science education. Res Sci Educ. 2018;48:1273-96. doi: 10.1007/s11165-016-9602-2.**

**36. Griethuijsen RAL, van Eijck MW, Haste H, den Brok PJ, Skinner NC, Mansour N, et al. Global patterns in students' views of science and interest in science. Res Sci Educ. 2015;45:581-603. doi: 10.1007/s11165-014-9438-6.**

9. I am a bit taken aback when the paper began to mention school fixed effects while the discussion has been about only moderation all along. I wonder if the authors are considering multilevel model initially and something didn’t work along the line, and it was scaled down to moderation analysis. – Thank you for your comment. While our article focuses on analyzing moderation effects, we incorporated school fixed effects to some models to control for all observed and unobserved differences between schools, which is a common and rigorous approach in education research. Initially, we also attempted multilevel modeling; however, given the relatively small variance between schools and some challenges with model identification in our sample, this approach proved less suitable. Consequently, we opted for fixed effects, which effectively remove between-school variation.

In this section, we are responding to each of the comments provided by Reviewer #1:

10. The authors should elaborate on how their findings extend or challenge existing literature, particularly in the Russian context. Thank you for your suggestion! We separated the introduction from the literature review and added relevant studies on subjective well-being (p. 5) and self-efficacy (p.8) conducted in Russia. These were the only studies which were conducted in Russia and were done rigorously enough to include them as part of the literature review section. As can be seen, there are a handful studies in general, and only a couple of studies conducted in upper elementary schools. We tried to emphasize this in the introduction on p. 3.
11. Consider discussing the implications of the high correlation between well-being subscales (r = 0.97) and between self-efficacy domains (r = 0.82), which may affect the interpretation of moderation effects. – Thank you for your comment. Given the very high correlations between well-being subscales (r = 0.97) and between self-efficacy domains (r = 0.82), we opted not to include both subscales or both domains simultaneously in the same models to avoid issues of multicollinearity. By modeling these constructs separately, we aimed to provide clearer, more reliable estimates of their distinct associations and potential moderation effects. We added this text on p. 12.
12. The introduction could be more concise, with a sharper focus on the research gap and the redundancy between the abstract and introduction could be reduced. – Thank you. The introduction was revised based on the suggestions (p. 3).

    The manuscript is suitable for publication pending minor revisions to enhance clarity and contextual framing. The authors are encouraged to:

Clarify the theoretical contribution of the study. – Thank you. We did it in the introduction on p. 3.
Expand on the practical significance of small effect sizes. – We highlighted it in the implications for practice on p. 25.
Streamline the introduction for conciseness. – thank you! The article now has the separate concise introduction (p.3) and literature review (4-8) sections.

We would like to thank Reviewers 1, 3, and the editor once again for your time and effort. We really appreciate it.

Sincerely,

Authors
