# Supplementary material for: Moderation analysis of subjective well-being, self-efficacy, and academic performance of 4th grade children in Russia
Source: PLoS One. 2026 Feb 2;21(2):e0341318. doi: 10.1371/journal.pone.0341318 (PMC12863477; doi:10.1371/journal.pone.0341318)
Supplement: Attachment — Submitted filename: Response to Reviewers_3.docx [file pone.0341318.s008.docx]

Letter of Revisions

This letter of revisions accompanies the revised manuscript *Moderation analysis of subjective well-being, self-efficacy, and academic performance of 4^th^ grade children in Russia* in response to the Reviewer’s 4 feedback as well as to editorial comments. We would like to express our deepest gratitude to anonymous reviewers who provided very detailed and constructive feedback on this paper. This letter of revisions provides the detailed response to each comment.

Based on the feedback, we revised the abstract, created separate introduction and literature, and highlighted the research gaps, novelty, and scientific significance of this study in the introduction and discussion sections. All the revisions to the text are represented in the blue font.

In this section, we are responding to each of the comments provided by Reviewer 4:

1. **Abstract:**

- The abstract should include research gap. – Thank you for your feedback. The research gap is added on p.1
- The abstract could more explicitly highlight the practical implications of the findings (e.g., how teachers or policy makers might use self-efficacy interventions). – Thank you. The implications are provided on p. 2
- The theoretical contribution—how this study extends existing literature—should be articulated more clearly. – Thank you. The theoretical contribution is added on p. 2
- A statement about the study design type (cross-sectional within a longitudinal project) could improve transparency. – Thank you. A phrase about the study design was added (p. 1).

2. **Introduction:**

- The opening paragraphs could be streamlined to avoid philosophical tone and improve focus. – Thank you for your suggestion. We tried to revise accordingly to avoid the philosophical tone. (pp. 3-4)
- Greater emphasis on why moderation (not mediation) was chosen theoretically would strengthen conceptual coherence. – Thank you for your suggestion. We tried to emphasize the moderation effect at the bottom of p.3.
- The contribution to the Russian educational policy or classroom practices could be articulated. – Thank you for your suggestion. We added a sentence about contributions at the end of the introduction section (p. 4).

3. **Literature Review:**

- The review could include more recent Russian or Eastern European studies to strengthen contextual grounding. - Thank you for your suggestion! Available well-being research from other Eastern European countries mostly focuses on older children (secondary school) (Mihojević et al., 2023; Simic et al., 2024) or even university students (Puiu et al., 2024).These studies are not directly related to the topic of present paper as they consider issues such as inequality (mostly focusing on SES; Mihojević et al., 2023; Simic et al., 2024), physical activity (Janda et al., 2025; Cefai at al., 2025) and others. However, we managed to find several studies looking into self-efficacy, which we added on p.7 (the paragraph in the blue font). We also
- Simić, N., Jović, S., & Petrović, D. (2024). Wellbeing of secondary school students from Serbia: The role of gender, socioeconomic status and ethnic background. In International Psychological Applications Conference and Trends (InPACT 2024), Book of proceedings (pp. 56-60). inScience Press.
- Mihojević, J., Simić, N., & Petrović, D. (2023). The role of gender and socioeconomic status in secondary school academic achievement and wellbeing. *Towards a more equitable education: from research to change-Book of Proceedings*, 36-43.
- Puiu, S., Udriștioiu, M. T., Petrișor, I., Yılmaz, S. E., Pfefferová, M. S., Raykova, Z., ... & Marekova, E. (2024, July). Students’ well-being and academic engagement: a multivariate analysis of the influencing factors. In *Healthcare* (Vol. 12, No. 15, p. 1492). MDPI.
- Janda D, Dygrýn J, Chmelík F, Rubín L, Juřicová T, Vorlíček M, Vencálek O, Gába A. Transitions of 24-H Movement Behaviour Profiles From Schooldays to Weekends and Their Associations With Health-Related Quality of Life and Well-Being in Czech Adolescents. Child Care Health Dev. 2025 Jul;51(4):e70121. doi: 10.1111/cch.70121. PMID: 40576241; PMCID: PMC12203759.
- Cefai, C., Barrado, B., Gimenez, G., & Cavioni, V. (2025). Adolescents’ Life Satisfaction, Physical Activity, and the Moderating Role of Gender: A Cross-Country, Multilevel Analysis in 64 Countries. *Children*, *12*(10), 1375. https://doi.org/10.3390/children12101375
- A conceptual model or diagram could visually depict hypothesized relationships and moderation pathways. – Thank you! We added a diagram in Figure 1 on p. 10.
- Greater critical synthesis (rather than descriptive summary) would highlight inconsistencies and justify hypotheses more strongly. - Thank you for your suggestion! We tried to revise the literature review and synthesize the literature. We also added the Current study section (p. 9), where we provide a synthesis of reviewed studies, articulate the research problem, and justify our study.

4. **Methodology:**

- Justify the choice of listwise deletion with a brief mention of sensitivity analysis (even a note that results were robust would help). Thank you for your comment. We added the following sentence on p. 11: “The listwise deletion was chosen since the sensitivity analyses indicated that the main results were robust to this choice.”
- Provide sampling frame size and how schools were proportionally selected to improve replicability. Thank you for your comment. We added information to the description of the sample on p. 11
- Mention socio-economic variables or regional educational indicators, if available, as potential covariates. – Thank you for this suggestion. Unfortunately, we don’t have those data for this dataset.
- Clarify that the reliability values below 0.70 (reading test) are acceptable within exploratory contexts, but indicate implications for interpretation.– Thank you for this comment. We specified this limitation in the methods section on p. 12. Also, we added a sentence in the limitations section (pp. 22-23) and called for using more robust instruments measuring reading skills in future research.

5. **Data analysis:**

- Consider testing the moderation using interaction plots or simple-slope analyses to visualize effects. – Thank you for this suggestion. The simple-slope plots were created for the whole sample and included in Appendix A on pp. 30-31.
- Include effect-size measures (e.g., ΔR² for moderation). - – Thank you for this suggestion. The effect size ΔR² for moderation was added in Tables 2-7 and included where appropriate in text of the results sections.
- Provide rationale for using fixed-effects instead of hierarchical linear modeling (though this is briefly justified, adding intra-class correlation coefficient would reinforce the decision). – Thank you for your suggestion. We added the following footnote on p.13 “The intra-class correlation coefficient (ICC), estimated from random-intercept null models, was 0.375 for math scores and 0.115 for reading scores, indicating substantial and moderate between-school variance, respectively. Although this suggests clustering at the school level, particularly for math scores, the primary analyses focused on models with school fixed effects to facilitate interpretation of moderation effects at the individual level. The use of school fixed effects is a common and rigorous approach in education research, as it controls for observed and unobserved differences between schools.”

6. **Results:**

- Tables could be condensed (e.g., combine similar models) for better readability. – Thank you for this suggestion. We condensed the tables on pp. 15-16.
- Include brief narrative summaries of main findings below tables to reduce cognitive load. - Thank you for this suggestion. We reorganized the tables, so they appear next to the text of the results, describing the estimates from the tables. We hope this has improved the readability of the results.
- Highlight practical significance of small effect sizes (β ≈ 0.05–0.10). – Thank you for this suggestion. We explicitly state the effect sizes are rather small in the discussion section; however, we draw readers’ attention to the role of self-efficacy and how teachers, policy makers, and other stakeholders should invest into developing self-efficacy of elementary school students in implications for practice on p. 24.

7. **Discussion:**

- Expand on socio-cultural explanations for gender differences (e.g., Russian classroom norms, parental expectations). – Thank you. We expanded on these explanations on p. 22.
- Explore implications for teacher practices (feedback, self-efficacy building) more deeply. – Thank you. We added this information on p. 24.
- Discuss potential reciprocal causation (academic success enhancing well-being) as a limitation. – Thank you for your comment. We added this information in the future research section on p. 23.

Editorial Comments

1. Streamlining the introduction and emphasizing the study’s theoretical novelty. – Thank you. We revised this part on pp. 3- 4.
2. Adding practical implications of findings for educators and curriculum planners. – Thank you. We added this information both in the abstract and implications for practice sections.
3. Improving table presentation and discussion clarity. – We revised the tables and reporting of the results.
4. Ensuring citation formatting aligns strictly with journal guidelines. – Thank you for your observation. We revised and edited the reference list in accordance with the Vancouver citation style.

We would like to thank Reviewer 4 and the editor once again for your time and effort. We really appreciate it.

Sincerely,

Authors
