# Supplementary material for: Moderation analysis of subjective well-being, self-efficacy, and academic performance of 4th grade children in Russia
Source: PLoS One. 2026 Feb 2;21(2):e0341318. doi: 10.1371/journal.pone.0341318 (PMC12863477; doi:10.1371/journal.pone.0341318)
Supplement: Attachment — Submitted filename: Reviewer Comments - PONE-D-23-27190_R2.docx [file pone.0341318.s005.docx]

The research is to examine the role of subjective well-being and self-efficacy in predicting academic achievement in math and reading among elementary school students in Russia. After careful review, here are my comments:

1. **Abstract:** The abstract effectively introduces the aim of the study, which is to explore how subjective well-being and domain-specific self-efficacy predict academic achievement in mathematics and reading among elementary school students in Russia. . However, the abstract would benefit from being more concise. Specific numerical results (e.g., strength of relationships, effect sizes) are missing, and the conclusion is vague. Including a sentence on practical implications could enhance its scholarly value and clarity.
2. **The keywords:** The keywords selected, “subjective well-being,” “self-efficacy,” “mathematics,” “reading,” “elementary school,” and “moderation,” are broadly relevant to the study’s scope. However, the inclusion of terms like “gender differences” and “academic achievement” would strengthen discoverability in academic databases.
3. **Introduction:** The introduction presents a compelling rationale for the research by highlighting the growing emphasis on students’ well-being alongside academic achievement. It provides a well-synthesized background on subjective well-being and self-efficacy, referring to both international and Russian educational contexts. The discussion of prior inconsistent findings helps justify the current research questions. Nevertheless, the introduction would benefit from improved logical flow and more emphasis on the novelty of this study, namely, the focus on elementary students in Russia using moderation analysis. Additionally, some opening paragraphs are slightly philosophical and could be tightened to maintain academic focus.
4. **Literature Review:** Although not presented as a distinct section, the literature review is embedded throughout the introduction and early pages. It provides a good overview of key concepts, such as subjective well-being and domain-specific self-efficacy, and discusses findings from major meta-analyses. The review includes important international sources and situates the study within the OECD’s broader educational goals. However, there is a limited critical analysis of Russian-specific literature, and some referenced studies are outdated or not fully detailed (e.g., missing author names). Structuring this into a standalone literature review section with clearer subsections and more recent sources would improve the depth and clarity of the theoretical foundation.
5. **Methodology:** The methodology section is detailed and logically structured. The study design is part of a longitudinal project, although the current data is cross-sectional. Participants were drawn from 40 schools in central Russia, with a sample size of 1,962 fourth-grade students. The use of well-validated instruments to assess well-being and self-efficacy is commendable, and psychometric properties are reported. The statistical approach, including OLS regression and moderation analysis with interaction terms, is appropriate and carefully explained. However, **justification for using listwise deletion for missing data instead of more robust methods (like multiple imputation) is lacking**. Also, the rationale for not testing both types of self-efficacy in the same model due to high correlation could be better supported with variance inflation factor (VIF) data. Additional information about the sampling process and school selection would strengthen transparency.
6. **Discussion:** The discussion section appropriately interprets the study’s findings and connects them to prior research. It confirms that while subjective well-being is weakly associated with academic outcomes, self-efficacy plays a stronger and more consistent role. The authors provide insightful commentary on gender differences, noting that the moderation effect of self-efficacy is only significant for girls in math. They also suggest that fourth-grade children may not yet have fully developed cognitive abilities to assess their self-efficacy accurately. However, the discussion could be improved by integrating more socio-cultural context into gendered perceptions of math and reading. Additionally, the potential influence of classroom practices and teacher feedback on self-efficacy is mentioned in limitations but not explored deeply in the interpretation of findings.
7. **References:** The references cited are mostly recent and relevant, including key meta-analyses, foundational theories, and empirical studies on self-efficacy and well-being. However, there are inconsistencies in citation formatting, and some references are incomplete (e.g., anonymous or missing authors). The inclusion of more Russian-language or context-specific studies would enrich the regional relevance of the paper.
